# Native sparse attention: co-designing algorithms and hardware for practical long-context efficiency

**DOI:** 10.1093/nsr/nwag212

**Published:** 2026-04-07

**Authors:** Jingyang Yuan, Ming Zhang

**Affiliations:** Key Laboratory for Multimedia Information Processing, School of Computer Science, Peking University, China; Key Laboratory for Multimedia Information Processing, School of Computer Science, Peking University, China

Long-context modeling has become increasingly important for large language models, enabling applications such as comprehensive document analysis, extended reasoning chains and multi-turn dialogue systems. Recent models including OpenAI’s o-series, DeepSeek-R1 [1] and Gemini 2.5 Pro [2] have demonstrated the value of processing long sequences with tens of thousands of tokens. However, the quadratic complexity of standard attention mechanisms [3] creates substantial computational bottlenecks. For 64k-length sequences, attention computation accounts for 70%–80% of total processing time, highlighting the need for more efficient approaches to reduce computational requirements while maintaining model performance.

Sparse attention represents a promising direction for addressing these computational challenges, leveraging the observation that softmax attention patterns often exhibit natural sparsity, whereby only a small subset of query-key pairs contribute significantly to the output. However, realizing the full potential of sparse attention faces two main challenges. First, many sparse attention methods do not align well with modern hardware architectures. Deep learning back ends, particularly those with Tensor Cores, are designed for dense, blockwise operations with high arithmetic intensity. Sparse patterns that involve irregular memory access or random token selection often fail to utilize these hardware capabilities effectively, limiting practical speed-up despite theoretical computational reductions. On the other hand, optimization requirements differ across computational phases: training and prefilling stages are typically compute-bound, while auto-regressive decoding is constrained by memory bandwidth due to the need to load large key-value caches.

Second, most sparse attention methods are designed primarily for inference, creating a training-inference divide that limits their effectiveness. These approaches typically rely on heuristic importance scoring or require auxiliary loss functions that add computational overhead. Without native training support, they cannot learn sparse patterns that are optimally suited to the model’s representational requirements, often resulting in performance degradation compared with full-attention models.

Existing sparse attention methods have adopted various design strategies with distinct trade-offs. Fixed-pattern methods, such as StreamingLLM [4], employ predefined local-window and global-token structures, ensuring hardware-predictable access patterns but lacking content-adaptive flexibility. Dynamic retrieval approaches, including InfLLM [5] and Quest [6], perform inference-time top-*k* key-value block selection without retraining the base model, enabling deployment on existing full-attention checkpoints while forgoing training-time sparsity adaptation. Block-level selection methods such as MoBA [7] integrate sparsity into training through Mixture-of-Experts-style routing over context blocks, achieving lower complexity with hardware-friendly coalesced access. Across these approaches, a central design consideration persists: coarse-grained block-level sparsity aligns with GPU memory hierarchies but may overlook fine-grained inter-token dependencies, whereas token-level selection captures more precise attention patterns at the cost of irregular memory access that reduces hardware throughput. The Native Sparse Attention (NSA) framework addresses this tension through co-designed algorithms and hardware implementations.

**Figure 1.**
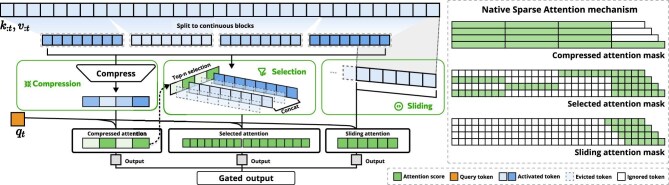
Overview of NSA’s architecture. Left: from top to bottom, the framework processes input sequences through three parallel attention branches: for a given query, preceding keys and values are processed into compressed attention for coarse-grained patterns, selected attention for important token blocks and sliding attention for local context. Right: top-*n* selection visualization of the different attention patterns produced by each branch. Green areas indicate regions where attention scores need to be computed, while white areas represent regions that can be skipped.

NSA [8] addresses these limitations through a co-designed approach that integrates algorithmic innovation with hardware-conscious implementation. As illustrated in Fig. 1, the method employs hierarchical sparse processing with three complementary pathways: compression for global context, selection for critical details and sliding windows for local patterns. The outputs of these three branches are fused through learned scalar gates at each query position, producing a weighted combination that enables the model to dynamically balance coarse global context, fine-grained critical detail and local continuity within a single forward pass. This design maintains both efficiency and representational completeness while avoiding the computational overhead of auxiliary losses.

NSA achieves hardware alignment through two design choices that leverage GPU memory characteristics. First, the selection branch operates at block granularity: the top-*k* selection set indexes entire contiguous key-value blocks, and the kernel sequentially loads these blocks into SRAM, exploiting the substantially higher GPU throughput for contiguous block access compared with random index reads. Second, the kernels adopt a group-centric data-loading strategy aligned with Grouped Query Attention (GQA): since all query heads within a GQA group share the same sparse key-value blocks, the kernel loads each block once into SRAM for the entire group, eliminating redundant memory transfers and sustaining high arithmetic intensity. The compression branch processes tokens in fixed-size segments via a learned per-block projection, maintaining full compatibility with the FlashAttention-2 pipeline. Together, these implementation choices ensure that NSA’s theoretical sparsity translates into proportional reductions in memory bandwidth consumption and practical wall-clock speed-up across training and inference phases.

Experimental validation on a 27B-parameter model demonstrates NSA’s effectiveness across multiple evaluation dimensions. NSA matches or exceeds full-attention performance on general benchmarks while showing improved performance in reasoning tasks. Long-context evaluation shows perfect accuracy in the needle-in-a-haystack [9] test and improved performance on multi-hop reasoning tasks, indicating that hierarchical sparse attention maintains both global awareness and local precision. Measured on NVIDIA H800 GPUs [8], NSA achieves up to 9$\times$ forward speed-up, 6$\times$ backward speed-up and 11.6$\times$ decoding acceleration at 64k context length, demonstrating practical performance improvements arising from theoretical sparsity.

The co-design principles of NSA have been further advanced through DeepSeek Sparse Attention (DSA), integrated into DeepSeek V3.2 [10]. DSA introduces targeted adaptations to NSA’s framework to enable efficient online serving at production scale. A notable design distinction concerns the indexer: in NSA, the model learns to identify important tokens through end-to-end sparse training, allowing the selection mechanism to co-evolve with the model’s representational capacity. In DSA, a dedicated lightweight Lightning Indexer is trained to approximate the distribution of the main attention branch via KL-divergence supervision, explicitly distilling the full-attention signal into a fast, low-cost index. This supervised approximation strategy provides a means of achieving high-quality token selection in serving scenarios where the indexer must operate under strict latency constraints. A second key adaptation concerns the training regime: rather than training from scratch with native sparsity as in NSA, DSA employs two-stage continued pre-training on an existing full-attention backbone (DeepSeek V3.1), followed by joint sparse training of all parameters. Together, NSA and DSA illustrate complementary approaches to hardware-efficient sparse attention: autonomous pattern learning through native training versus explicit distribution approximation through supervised indexing, both grounded in hardware-conscious co-design.

These results suggest several principles for practical sparse attention design. Algorithm development should consider hardware characteristics, pursuing balanced arithmetic intensity across computational phases rather than focusing solely on theoretical complexity reduction. Training and inference optimization benefit from unified design rather than distinct treatment; this unified approach enables end-to-end learning of optimal sparse patterns. Hierarchical information processing appears to provide more robust performance than single-sparsity strategies, allowing different pathways to specialize in complementary aspects of context modeling.

NSA suggests several directions for future research. Dynamic sparse-pattern learning may further optimize attention allocation based on input characteristics. Cross-modal applications could extend hierarchical sparse attention beyond text to vision and multimodal contexts, where spatial locality naturally complements the sliding-window branch. Co-evolution with emerging hardware trends offers further opportunities: as memory bandwidth, compute density and low-precision quantization continue to advance, sparse attention is increasingly well-positioned to exploit these improvements—higher compute density amplifies the benefit of reducing unnecessary attention operations. Furthermore, as models scale to larger parameter counts and longer contexts, the absolute computational savings from sparsity grow proportionally, making hardware-aligned sparse attention an increasingly important component of sustainable large-scale training and inference.

However, several challenges remain to be addressed. Scaling to sequences beyond 1M tokens requires new approaches to sparse-pattern design and memory management. Integration with other efficiency techniques such as Mixture-of-Experts requires careful consideration of computational and communication trade-offs. Theoretical analysis frameworks for understanding sparse attention’s representational capabilities require further development.

In conclusion, NSA represents a shift from treating sparsity as a post-hoc optimization to incorporating it as a fundamental design principle. By co-designing algorithms and hardware optimizations, NSA demonstrates that the theory-practice gap in sparse attention can be addressed, providing new possibilities for efficient long-context modeling at scale. As large language models continue to extend context length and computational requirements, this integrated approach offers a framework for sustainable progress in the field.
